# A Study of the Etiology of Referred Otalgia

**Published:** 2012

**Authors:** Mohammad Hosein Taziki, Nasser Behnampour

**Affiliations:** 1 *Department of Otorhinolaryngology, Golestan Universityof Medical Sciences, Gorgan, Iran.*; 2 *Hematology & Oncology Research Center, Golestan University of Medical Sciences, Gorgan, Iran.*

**Keywords:** Cases, Earache, Pain, Referred

## Abstract

**Introduction:**

Otalgia is one of the complaints which may occur at any age. The etiology of the pain may be in the ear, structures around the ear or other head and neck structures. This is caused by the complex nervous connections in the head and neck areas, the ear, the pharynx and the nose. Since understanding the etiologies of referred otalgia can help in the assessment and treatment of the disease, this research was conducted to identify the etiologies of referred otalgia in patients visiting the ENT Clinic in Gorgan, Iran.

**Materials and Methods:**

This prospective research was conducted on patients who visited the ENT Clinic with an earache, but in initial assessments the ear was normal. Patients’ data consisting of sex, age, complaint, the inflicted side, physical findings in the ear, the nose, the throat and head and neck were recorded in a questionnaire. These data were then analyzed with SPSS software.

**Results:**

Of 770 patients with otalgia, 94 patients (12.2%) had referred otalgia. Of these patients 27.7% were men and 72.3% were women. The most common etiology of referred otalgia was dental problems (62.8%), and one patient who was being treated for pharyngitis had carcinoma of the base of the tongue. In 47.8% of cases the pain was in the left ear, in 43.4% in the right ear, and in 8.7% it was bilateral.

**Conclusion:**

In view of the fact that a significant proportion of the patients who complained of otalgia had no pathologies in the ear, thorough physical examination in adjacent structures especially teeth should be performed and malignancies should be considered as a possible etiology of otalgia.

## Introduction

Otalgia can originate from pathologies inside the ear (primary otalgia) or can be a referred pain originating from outside the ear (referred otalgia) (1).

If the ear is found normal in the examination, Otalgia will be considered referred pain. In many patients who have normal ears, the origin of the pain should be sought much further away (2). The ear receives its sensory nerves from six sources, and several other head and neck structures share a common nerve supply (3). Thus, pathologies occurring in the neural network of cranial nerves V, VII, IX, and X and cervical spinal nerves C2 and C3 can be considered as possible etiologies of referred otalgia (4). Children tend to suffer from primary otalgia, while adults tend to suffer from referred otalgia (5).

In one study, the most common etiology of referred otalgia was found to be dental athologies, accounting for 50% of the cases (6).

In another study, the incidence of otalgia was found to be 33% in patients suffering from the carcinoma of the base of the tongue (7).

The pathologies of cervical vertebrae have also been identified as causing referred otalgia (8). Bell's palsy can lead to otalgia, which is considered referred otalgia as the ear examination is normal (9). An enlarged styloid process (11) and metastases to the pharynx (12) have been identified as possible causes of referred otalgia. In another study, a patient complaining from otalgia was later found to suffer from nasopharyngeal carcinoma (12).

As the muscles of the middle ear have the same embryological origins as those of the face, pains originating from those areas can be felt in the throat. These and the malfunction of temporomandibular joint can also cause referred otalgia (13). If physical examinations and case histories do not lead to conclusive diagnoses, it is recommended that a course of symptomatic treatment be administered, and upon failure of this, further systematic examinations be conducted (13).

As there is no simple, single algorithm for identifying referred otalgia, and as the areas supplied by the above-mentioned nerves are extensive and as late diagnoses can lead to irreparable consequences (5), the present study was conducted to identify the etiologies of referred otalgia and to underline the need for increased attention to the dangerous etiologies.

## Materials and Methods

This study was conducted in 2009 and 2010 on patients visiting the ENT clinic. The patients underwent thorough examinations, and those who had a normal ear examination were selected for the study. The examinations covered the teeth, the temporomandibular joint, the nose, the sinuses, and head and neck areas, and when necessary, examinations included direct and indirect laryngoscopy and biopsy.

Those who had previously undergone tonsillectomy or other head and neck surgeries and had later developed otalgia were excluded from the study. Patients’ data including age, sex, season of the disease development, the afflicted side and the cause of the otalgia were recorded. In view of the wide spectrum of the etiologies of referred otalgia, when necessary, consultations were made with other specialists such as dentists. The collected data were then statistically analyzed with the SPSS software.

## Results

Out of 770 patients complaining from earaches, 94 (12.2%) suffered from referred otalgia, in eight of whom pathologies were simultaneously found both in the ear and in the adjacent structures. In the referred otalgia patients, 72.3% were women and 27.7% were men.

In this study the etiologies of referred otalgia were investigated, the most frequent of which was toothache (62.8%). Other etiologies included pharyngitis (24.5%), inflammation of the temporomandibular joint (8.5%), sinusitis (2.1%), pharyngeal abscess (1.1%) and Bell’s Palsy (1.1%) (Table 1). In one female patient who had long been receiving treatment for pharyngitis, there was a supraglottic tumor, involving the base of the tongue, which had surface exudates, and the biopsy confirmed SCC (Table 1). In this study, the etiology of referred otalgia was also studied in terms of sex (Table 1).

**Table 1:** Incidence of referred otalgia in terms of sex

**Sex**	Female Number (%)	Male Number (%)	Total Number (%)
**Etiology**			
Dental Problems	42(61.8)	17(65.4)	59(62.8)
Pharyngitis	18(26.4)	5(19.2)	23(24.5)
Temporomandibular Joint	6(8.8)	2(7.7)	8(8.8)
Sinusitis	0(0.0)	2(7.7)	2(2.1)
Bell’s Palsy	1(1.5)	0(0.0)	1(1.1)
Pharyngeal Abscess	1(1.5)	0(0.0)	1(1.1)
Total	68(100)	26(100)	94(100)

Also the inflicted side and the etiology were studied. In 47.8% of cases, the left side was inflicted, in 43.4% the right side,and in 8.7% both sides.

Also the etiology was studied in terms of age groups (Table 2,3).

**Table 2:** Incidence of referred otalgia in terms of the afflicted side

**Afflicted Side**	Left Number (%)	Right Number (%)	Bilateral Number (%)
**Etiology**			
Dental Problems	32(55.2)	22(37.9)	4(6.9)
Pharyngitis	9(39.1)	10(43.5)	4(17.4)
Temporomandibular Joint	2(26.8)	5(71.4)	0(0.0)
Sinusitis	0(0.0)	2(100.0)	0(0.0)
Bell’s Palsy	1(100.0)	0(0.0)	0(0.0)
Pharyngeal Abscess	0(1.5)	1(100.0)	0(0.0)
Total	44(47.8)	40(43.5)	8(8.7)

**Table 3:** Incidence of referred otalgia in terms of the afflicted side

**Age (years)**	≤ 5 Number (%)	6-20 Number (%)	21-35 Number (%)	36-50 Number (%)	> 50 Number (%)
**Etiology**					
Dental Problems	0(0.0)	17(63.0)	25(67.6)	13(54.2)	4(80.0)
Pharyngitis	1(100.0)	8(29.6)	8(21.6)	5(20.8)	1(20.0)
Temporomandibular Joint	0(0.0)	1(3.7)	2(5.4)	5(20.8)	0(0.0)
Sinusitis	0(0.0)	1(3.7)	1(2.7)	0(0.0)	0(0.0)
Bell’s Palsy	0(0.0)	0(0.0)	1(2.7)	0(0.0)	0(0.0)
Pharyngeal Abscess	0(0.0)	0(0.0)	0(0.0)	1(4.2)	0(0.0)
Total	1 (100.0)	27(100)	37(100)	24(100)	5(100)

## Discussion

Otalgia is an unpleasant experience causing people of different ages to visit doctors. Since the ear sensory nerve supply originates from different nerves, pathologies of different head and neck structures can manifest themselves as otalgia, causing patients to seek medical help. As the patient may well be unaware of any conditions outside his/her ear or of the fact that the cause may be outside the ear, otalgia is the chief complaint of the patient. When dealing with otalgia patients, taking the case history is the first step, followed by physical examinations, and ultimately and if necessary, paraclinical measures and consultations with specialists from other fields.

In case of normal ear examination, otalgia is defined as referred otalgia, and in the case of ear pathologies, it is defined as primary.

In our study, 770 patients suffered from otalgia, 12.2% of whom had the referred type. In a study on 143 otalgia patients by Kiakojoori et al at the Shahid Beheshti Hospital, Babol, Iran, in 1999, the incidence of referred otalgia was reported at 46% (15).

In another study on 300 otalgia patients by Behnood et al in Hamedan, Iran, the incidence of referred otalgia was reported at 30.6% (15). The results of these last two studies are consistent with those of the present study.

In Neilan’s study, otalgia has been reported differently in terms of age, i.e. children tended to suffer from the primary type while adults tended to suffer from the referred type (5). Thus, there is a slight discrepancy compared with our results.

In this study, 72.3% of the patents were women and 27.7% were men. In Kiakojoori et al’s study, these figures were 40% and 60% respectively, which are consistent with our findings.

According to our study, the most frequent etiology of referred otalgia was found to be toothache (62.8%), followed by pharyngitis (24.5%), the temporomandibular joint (8.5%), sinusitis (2.1%), pharyngeal abscess (1.1%) and Bell’s palsy (1.1%). It should be added that in one patient, initially admitted for pharyngitis, the final diagnosis was supraglottic SCC. In Kiakojoori’s study, in 45% of the cases, toothaches were the cause of the referred otalgia (15).

In Behnoud et al’s study, the most frequent etiology was reported to be the temporomandibular joint (16). In Kim’s study, toothache accounted for most cases of referred otalgia (50%). In our study, there was only one patient with supraglottic carcinoma.

In Dally’s study, there was one case of lower-extremity liposarcoma metastasis to the pharynx which had manifested itself as sore throat and otalgia (11).

In Reiter’s study, too, there was a case of nasopharyngeal carcinoma manifested as referred otalgia (12).

In Mulwafu’s study on 17 patients suffering from the carcinoma of the base of the tongue, 33% of the cases suffered from referred otalgia (7).

Also, Han reported one case of Bell’s palsy, first manifested itself as otalgia with no other findings in the examinations (9).

In Kiakojoori et al’s study, 6% of the etiologies of referred otalgia were reported as pharyngeal carcinoma, which was a higher rate than in our study (15).

## Conclusion

In this study, the rate of referred otalgia is lower than those in other studies. However, the high incidence of referred otalgia with dental etiologies underlines the point that in addition to our thorough examinations of the ear, the pharynx, the nose and head and neck structures, we should pay attention to dental hygiene in patients with otalgia in order to identify diseases outside the ear. Further, as some malignancies manifest themselves as otalgia, this should be considered in diagnoses.
